# Supplementary material for: Identification of Pathogenic and Opportunistic Yeasts in Pigeon Excreta by MALDI-TOF Mass Spectrometry and Their Prevalence in Chon Buri Province, Thailand
Source: Int J Environ Res Public Health. 2023 Feb 11;20(4):3191. doi: 10.3390/ijerph20043191 (PMC9967633; doi:10.3390/ijerph20043191)
Supplement: Supplementary file 1 [file ijerph-20-03191-s001.zip › ijerph-2041665-supplementary.pdf]

International Journal of
Environmental Research and Public Health

MDPI

Supplementary Materials

Identification of Pathogenic and Opportunistic Yeasts in Pigeon Excreta by MALDI-TOF Mass Spectrometry and Their Prevalence in Chon Buri Province, Thailand

Rungnapa Nualmalang [1], Natthapaninee Thanomsridetchai [2], Yothin Teethaisong [1,3], Passanesh Sukphopetch [4] and Marut Tangwattanachuleeporn [1,3,*]

[1] Department of Medical Sciences, Faculty of Allied Health Sciences, Burapha University, Chonburi 20131, Thailand
[2] Department of Medical Technology, Faculty of Allied Health Sciences, Burapha University, Chonburi 20131, Thailand
[3] Research Unit for Sensor Innovation (RUSI), Burapha University, Chonburi 20131, Thailand.
[4] Department of Microbiology and Immunology, Faculty of Tropical Medicine, Mahidol University, Bangkok 10400, Thailand
* Correspondence: marutt@go.buu.ac.th; Tel.: +66 82 661 6354

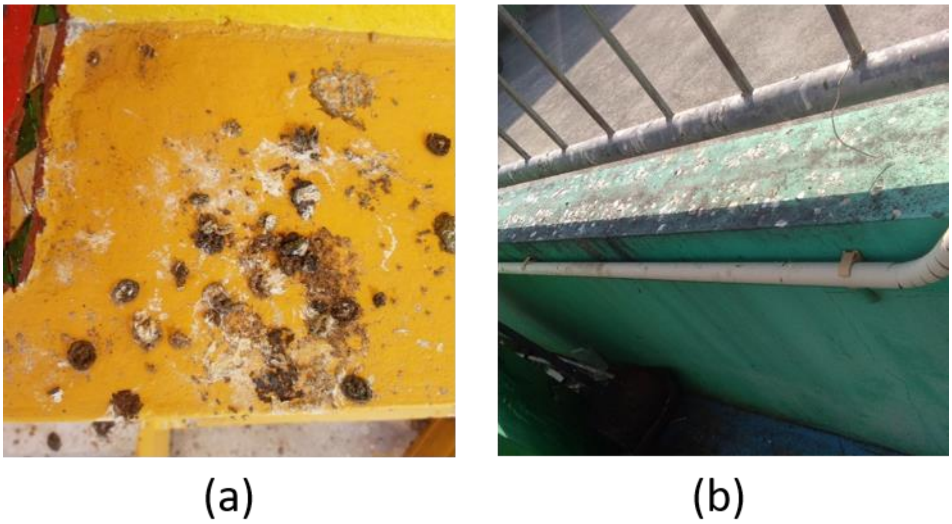

Figure S1. The difference between pigeon droppings (**a**) and Old world sparrows' feces (**b**).

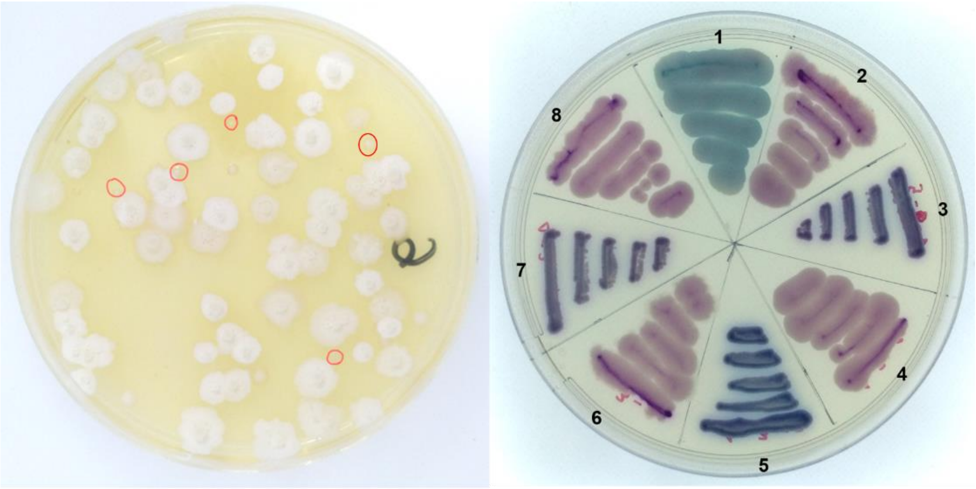

(a) (b)

Figure S2. Colony morphology of yeasts-like isolates that isolated from pigeon excreta on (**a**) SDA contain 0.4 g/L Chloramphenicol; (**b**) representative of distinct yeast species on CHROMagar medium. Red circles represent morphology of yeast-like isolates. 1 = *Lodderomyces elongisporus* BUU9; 2 = *Candida krusei* BUU11; 3 = *Candida tropicalis* BUU12; 4 = *Candida krusei* BUU14; 5 = *Candida tropicalis* BUU15; 6 = *Candida krusei* BUU16; 7 = *Candida tropicalis* BUU17; 8 = *Candida krusei* BUU8.
